# Effects of the SES NXT intervention on mental health and well-being for children of divorce

**DOI:** 10.1038/s41746-026-02638-x

**Published:** 2026-04-21

**Authors:** Gert Martin Hald, Daniel Bach Johnsen, Theis Lange, Andreas Nielsen Hald, Søren Sander, Camilla Stine Øverup

**Affiliations:** 1 Department of Public Health, University of Copenhagen, København K, Denmark; 2 School of Psychology, Deakin University, Melbourne, VIC Australia; 3 Department of Public Health, Aarhus University, Aarhus C, Denmark

**Keywords:** Diseases, Health care, Medical research, Psychology, Psychology

## Abstract

Parental divorce is common and linked to adverse mental health outcomes and reduced well-being in children and adolescents, yet digital interventions for this group remain scarce. This study reports on a randomized controlled trial of a digital intervention (SES NXT) for children and adolescents aged 3–17 experiencing parental divorce. Participants (*n* = 866) were randomized to either SES NXT (*n* = 449) or a waitlist control group (*n* = 417). At 12-week follow-up from baseline, the intervention group showed medium to large improvements across all primary and secondary mental health and well-being outcomes versus the waitlist control group, as measured by the Strength and Difficulty Questionnaire (SDQ). Primary outcomes included emotional symptoms, total difficulties, and impairment (Cohen’s (*d*) = 0.66–0.71, all *p’s* < 0.001). Secondary outcomes included conduct problems, hyperactivity, peer problems, and prosocial behavior (Cohen’s (*d*) = 0.47–0.56, all *p’s* < 0.001). Findings are discussed through the Divorce-Stress-Adjustment framework and within the Northern European (Danish) cultural context.

## Introduction

To most children and adolescents, divorce represents a significant disruption to their familial environment and a significant adverse life event, with a resulting drop in psychological and physical well-being and quality of life^1–5^. In this paper, we use the term “divorce” to refer to both juridical and non-juridical relationship dissolution.

In the short term, children and adolescents affected by divorce frequently experience heightened emotional problems^1,6,7^, behavioral difficulties^8^, declines in physical and mental health^7,9,10^ and educational declines^11^. Over the longer term, divorce can also have adverse consequences that extend into adulthood, such as poorer mental health, lower educational attainment, and worse labor market outcomes^11–14^. However, findings on these long-term effects are mixed and indicate substantial variation in how divorce affects children over time. This variation reflects the operation of multiple, interacting mechanisms of adjustment, including developmental, contextual, relational, and psychological mechanisms, that shape how children experience and adapt to family change^15^. Developmental stage, socioeconomic background, and time since the divorce are key among these, influencing both the expression and trajectory of adjustment processes to significant family change^16^. For example, younger children may display more externalizing or attachment-related responses, whereas adolescents often experience heightened internalizing symptoms and identity-related stress^17^. Similarly, socioeconomic instability or loss can amplify post-divorce strain, while economic and social resources may buffer against its effects^18^. Time since divorce also influences adaptation, with some difficulties diminishing as coping skills strengthen and as experience living within a divorced family increases, while others emerge later in response to new developmental or family transitions such as when a parent remarries or relocates farther away from the child.

Beyond these developmental and contextual influences, psychological and relational processes play a critical role. Children’s psychological resilience, expectations surrounding the divorce, and the quality of parent–child communication and bond interact dynamically with environmental and temporal factors to determine both vulnerability and recovery trajectories^8,19–23^. For example, high parental conflict, reduced consistency between homes, or diminished emotional availability can exacerbate stress, whereas positive parental cooperation and open communication can facilitate adjustment^24^.

This integrated view aligns with one of the most influential and used theoretical perspectives in the area, namely the Divorce–Stress–Adjustment Perspective (DSAP), which conceptualizes divorce not as a discrete event but as an evolving process in which outcomes are determined by the balance between stressors, individual capacities, and protective resources^18,25^. Stressors may include parental conflict, new living arrangements, declining financial resources, and the formation of new stepfamilies^26–28^, whereas protective mechanisms encompass strong parent–child relationships^29,30^, effective coping skills^31^, and the meanings children ascribe to the divorce^32^ (e.g., loss, gain, or healing). Taken together, these developmental, contextual, and psychosocial mechanisms illustrate that children’s and adolescents’ adjustment to divorce is best understood as a dynamic, multilevel process shaped by interacting systems of risk and protection across time.

Given that prevalence rates of divorce are high across all Western countries, usually ranging from 35-50%, divorce affects millions of children and adolescents annually^33^. In Denmark, where the current study is conducted, the annually divorce rate usually ranges between 40 and 45% and approximately 25% of all children and adolescents do not live with both of their biological parents, most often due to parental divorce^34,35^. Among both divorced families and professionals working with them, a valid question therefore remains how to best help children and adolescents of parental divorce cope with and navigate through the aftermath of the divorce. In this regard, digital interventions present a logical and promising solution for the “born digital” generations^36^ and have been called for^37^. Beyond their 24/7 accessibility and convenience compared to face-to-face options, they offer scalability, cost-effectiveness, minimal logistics, reduced (self)stigma, and the flexibility to be updated, expanded, and personalized as needed^15,38,39^.

In the divorce research field, research involving divorced adults shows that digital interventions can help mitigate well-known adverse post-divorce effects such as stress, anxiety, and depression, generally improve mental and physical health, and reduce sick days during the first 12 months following divorce^33,40,41^. Yet, despite this potential, digital interventions targeting children and adolescents have remained greatly underdeveloped and underexplored^15,42^. In fact, to the best of our knowledge, to date, only one digital intervention aimed directly at children and adolescents of divorced parents has been created and tested, namely the *Children of Divorce–Coping with Divorce* (CoD-CoD) program^43^. This online, cognitive-behavioral program for adolescents aged 11–16 years has demonstrated moderate short-term improvements in youth-reported mental health and adjustment outcomes in a U.S. randomized controlled trial (RCT) (*n* = 147; 4-week follow-up)^37,43^. Similarly, a second intervention is currently under development, but it also targets only a smaller age cohort of youth aged 9–12 (*n* = 144)^44^. Because divorce may affect children differently across developmental stages, and digital, cognitive, emotional, social, and behavioral capacities vary widely with age, it is essential to design interventions that can accommodate multiple age groups of children and adolescents.

The present study, therefore, investigates the efficacy of *SES NXT*, a newly developed, fully digital intervention designed for children and adolescents aged 3–17 years who have experienced parental divorce. Building on insights from CoD-CoD, SES NXT extends this earlier work by incorporating (a) a much broader developmental range supported by age-specific modules and user interfaces; (b) a social–emotional and mentalization-based framework emphasizing normalization, agency, and connectedness within family systems, rather than a solely cognitive-behavioral coping focus; and (c) the possibility of parental involvement to support engagement among the younger participants. Accordingly, SES NXT represents digital family support that aims to deliver early, scalable care across childhood and adolescence. Comprehensive details about the intervention are provided in the “Methods” section and in Øverup et al.^15^.

The primary aim of this evaluation is to determine whether SES NXT effectively enhances mental health and well-being among children and adolescents who have experienced parental divorce. To this end, we test three primary (H1–H3) and four secondary (H4–H7) hypotheses from the SES NXT study. The evaluation is based on a large-scale, pre-registered RCT involving Danish children and adolescents and their parents. The RCT includes an intervention group and a waitlist control group (WL), with assessments conducted at baseline (T1), 4 weeks post baseline (T2), and 12 weeks post baseline (T3). The selection of primary and secondary outcomes reflects SES NXT’s theoretical foundation and practical design. Developed within the theoretical framework of the DSAP, SES NXT primarily targets emotional adaptation and overall psychosocial adjustment through its emphasis on emotional literacy, normalization, coping, and agency, and on translating these competencies into constructive actions and behaviors. Accordingly, emotional symptoms, the Strength and Difficulty Questionnaire (SDQ) total difficulties score, and the SDQ impact score were chosen as primary outcomes, as they best capture the intervention’s intended impact on emotional regulation, general mental health, and the everyday impact of change. The additional behavioral and social domains, including conduct problems, hyperactivity, peer relations, and prosocial behavior, were designated as secondary outcomes to capture other potential changes arising from components of the intervention that focus more on social and behavioral functioning than on emotional functioning. This outcome hierarchy thus mirrors both the intervention’s developmental rationale and its action-oriented structure, which supports emotional understanding as a foundation for behavioral change and adaptive coping. Accordingly, in the following, we test the following hypotheses:

Hypothesis 1 **(H1):** The intervention group will report significantly fewer emotional symptoms than the control group at T3 (12 weeks) as measured by the SDQ emotional symptoms score.

Hypothesis 2 **(H2):** The intervention group will report significantly better mental health than the control group at T3 (12 weeks) as measured by a lower overall SDQ score.

Hypothesis 3 **(H3):** The intervention group will report significantly lower overall distress and impairment than the control group at T3 (12 weeks) as measured by a lower SDQ impact score.

**Hypotheses 4–7 (H4-7):** Compared to the control group, the intervention group will report significantly lower SDQ scores in terms of conduct problems (**H4**), hyperactivity/inattention (**H5**), and peer relationship problems (**H6**), and higher levels of prosocial behavior (**H7**) at T3 (12 weeks).

In addition, to contextualize the results, we compare the results from the three primary hypotheses (H1-3) with the corresponding national Danish norms of the SDQ and report on the participants’ perceived effects of their participation in the SES NXT project on problem reduction from study enrollment to study conclusion.

## Results

Table 1 provides the raw means and standard deviations for each SDQ outcome by intervention group. Table 2 provides the results of the test of group differences at T3 (i.e., 12 weeks follow-up) for all outcomes related to the study hypotheses (H1-7). The results of the unadjusted analyzes can be found in Supplementary Table 3. For the three primary study hypotheses (H1-3), we found a significant group differences at T3 with the intervention group reporting significantly lower scores on Emotional Symptoms (H1), Overall Total Psychological Difficulties (H2) and Overall Distress and Functional Impairment (H3) compared to the WL control group, with the magnitude of these differences being medium to large in effect size (Cohen’s (*d*) = 0.66–0.71, all *p*’s < 0.001). Similarly, for the secondary hypotheses (H4-7), we found significant group differences at T3 with the intervention group reporting significantly lower scores on Conduct Problems (H4), Hyperactivity/Inattention (H5), and Problems with Peers (H6) and significantly higher scores on Prosocial Behavior (H7) compared to the WL control group, with the magnitude of these differences being medium in effect size (Cohen’s (*d*) = 0.47–0.56, all *p*’s < 0.001). These results supported our primary and secondary hypotheses.

**Table 1:** Raw means and standard deviations for all SDQ outcome scores

Outcome measure	Group	M (SD)					
		T1		T2		T3	
Emotional symptoms	NXT	6.49	(3.03)	3.75	(1.93)	3.06	(1.79)
	WL	6.47	(3.15)	6.17	(3.20)	5.60	(3.02)
Total Score	NXT	17.78	(7.17)	11.37	(5.13)	10.20	(4.77)
	WL	17.91	(7.61)	17.68	(7.29)	17.05	(7.12)
Impact	NXT	3.47	(2.90)	0.93	(1.37)	0.66	(1.34)
	WL	3.34	(2.97)	3.05	(2.71)	2.80	(2.62)
Conduct problems	NXT	3.46	(2.15)	2.20	(1.70)	2.02	(1.52)
	WL	3.54	(2.20)	3.61	(2.10)	3.47	(2.07)
Hyperactivity/Inattention	NXT	4.86	(2.13)	3.53	(2.02)	3.33	(2.03)
	WL	4.82	(2.24)	4.69	(2.04)	4.81	(2.17)
Problems with peers	NXT	2.96	(2.08)	1.88	(1.76)	1.78	(1.69)
	WL	3.07	(2.07)	3.22	(1.98)	3.17	(2.00)
Prosocial behavior	NXT	6.21	(2.07)	7.11	(1.90)	7.43	(1.75)
	WL	6.25	(2.32)	6.11	(2.22)	6.12	(2.12)

T1 = baseline, T2 = 4-week follow-up, T3 = 12-week follow-up. *N*_intervention_ = 449, *N*_WL_ = 417.

**Table 2:** Tests of effectiveness of the intervention as defined by a test of group differences at T3 and a test of group differences over time

	Between-group difference at T3						Group*time effect	
Outcome measure	Mean diff.	(95% CI)		*z* value	*p* value	Cohen’s *d*	*Chi-Square*	*p* value
Emotional symptoms	1.99	(1.59;	2.39)	9.71	<0.001	0.66	110.92	<0.001
Total Score	5.12	(4.17;	6.06)	10.62	<0.001	0.71	107.41	<0.001
Impact	1.67	(1.33;	2.00)	9.69	<0.001	0.66	99.41	<0.001
Conduct problems	1.06	(0.81;	1.32)	8.08	<0.001	0.54	54.34	<0.001
Hyperactivity/inattention	1.03	(0.74;	1.31)	7.12	<0.001	0.47	62.44	<0.001
Problems with peers	1.07	(0.78;	1.35)	7.26	<0.001	0.49	44.09	<0.001
Prosocial behavior	−1.04	(−1.33;	−0.76)	−7.15	<0.001	0.46	52.40	<0.001

*N*_intervention_ = 449, *N*_WL_ = 417. T3 = 12-week follow-up. The results for the between-group difference come from Analysis of GEE Parameter Estimates (WL vs. intervention group), while the Group*time effect comes from a Type 3 GEE Analysis. These tests are standard output from SAS proc genmod; model / type3.

### Group x time effects

**Fig. 1:**
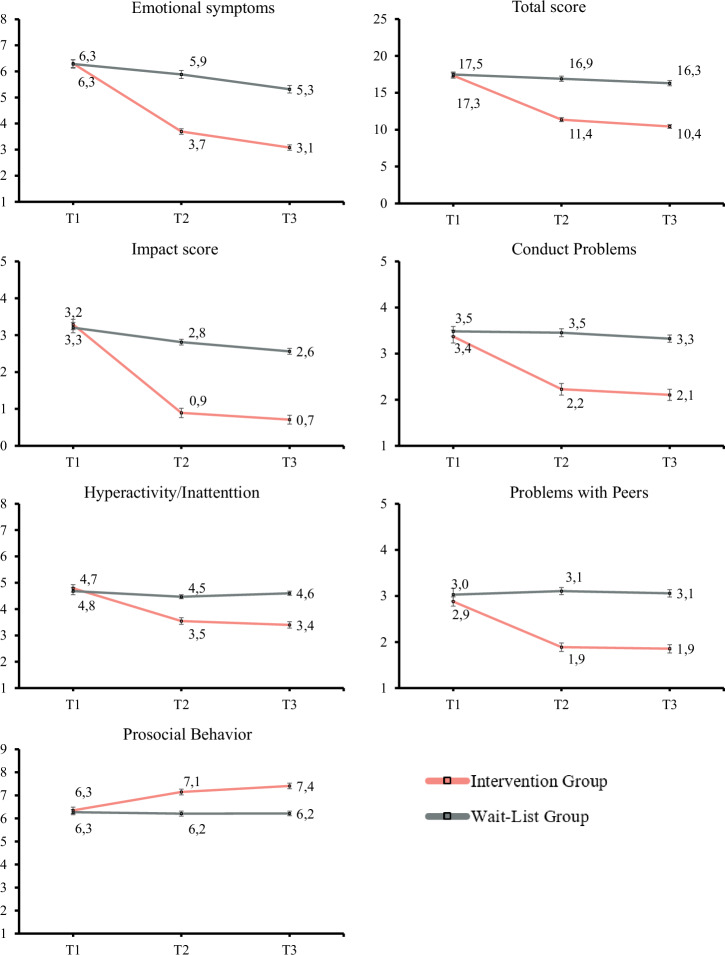
Change in scores over time for the intervention and WL group. The estimated means and associated standard errors (as bars) presented in the figure come from the analyses that examined a Group x Time effect and were estimated using an LSMEANS statement (in SAS *proc genmod*). Covariates (i.e., participants’ gender and age, parental gender and age, educational level, income, and parental time-varying mental health symptoms) were included in the analysis. T1 = baseline, T2 = 4-week follow-up, T3 = 12-week follow-up.

To test whether changes in scores between the intervention and the control group differ over time, we conducted a Group-by-Time test for each study outcome. As can be seen from both Table 2 and Fig. 1, for all outcomes, we found a significant effect, indicating that scores on the outcome measures varied by group over time. Further probing of these results (see Table 3) showed that there were no significant differences between the intervention group and the control groups on the outcome measures at baseline (T1), but that the scores between the intervention group and the control group differed at both T2 and T3, with the intervention group showing significantly greater improvements than the WL control group across outcomes. Differences between the intervention and WL control group were medium to large in size at T2 (Cohen’s *d* = 0.44–0.90) and at T3 (Cohen’s *d* = 0.55–0.96).

**Table 3:** Comparisons between intervention and control group at each time point

Group	Group	Time	*Estimate*	*St. Error*	*z value*	*p value*	*d*
Emotional symptoms							
Control group	Intervention group	1	−0.02	0.21	−0.09	0.932	0.01
Control group	Intervention group	2	2.19	0.18	12.12	<0.001	0.80
Control group	Intervention group	3	2.24	0.17	12.92	<0.001	0.86
Total score							
Control group	Intervention group	1	0.14	0.50	0.29	0.774	0.02
Control group	Intervention group	2	5.56	0.42	13.29	<0.001	0.87
Control group	Intervention group	3	5.87	0.40	14.71	<0.001	0.96
Impact							
Control group	Intervention group	1	−0.09	0.19	−0.45	0.651	0.03
Control group	Intervention group	2	1.92	0.14	13.32	<0.001	0.90
Control group	Intervention group	3	1.85	0.14	13.06	<0.001	0.88
Conduct problems							
Control group	Intervention group	1	0.11	0.14	0.78	0.434	0.05
Control group	Intervention group	2	1.23	0.13	9.61	<0.001	0.63
Control group	Intervention group	3	1.22	0.12	10.12	<0.001	0.66
Hyperactivity/inattention							
Control group	Intervention group	1	−0.11	0.16	−0.68	0.495	0.05
Control group	Intervention group	2	0.92	0.14	6.71	<0.001	0.44
Control group	Intervention group	3	1.20	0.14	8.45	<0.001	0.55
Problems with peers							
Control group	Intervention group	1	0.15	0.14	1.08	0.281	0.07
Control group	Intervention group	2	1.21	0.13	9.60	<0.001	0.64
Control group	Intervention group	3	1.20	0.13	9.60	<0.001	0.64
Prosocial behavior							
Control group	Intervention group	1	−0.08	0.15	−0.53	0.593	0.04
Control group	Intervention group	2	−0.94	0.14	−6.53	<0.001	0.44
Control group	Intervention group	3	−1.19	0.13	−9.15	<0.001	0.61

T1 = baseline, T2 = 4-week follow-up, T2 = 12-week follow-up.

To test whether differences in scores between the intervention and the control group differed by intervention age group, we examined a three-way interaction between time, group (intervention vs. WL group), and intervention age group (3–5, 6–8, 9–12, and 13–17). As can be seen in Table 4, this three-way interaction was not statistically significant across all outcomes, indicating that those in the intervention group endorsed significant improvements as compared to the WL group at each time point, regardless of intervention age group. Supplementary Fig. 2 and Supplementary Table 4 demonstrate that there were no significant differences at T1 between the intervention and WL group for all age groups, while at T2 and T3, there were significant differences between the intervention and WL groups for all age groups, with the exception of Hyperactivity/Inattention at T2, where age 3–5 and age 6–8 did not differ, and Prosocial Behavior at T2, where age 9–12 did not differ, based on Bonferroni-adjusted thresholds.

**Table 4:** Tests of differences between intervention and WL group by intervention age group over time (3-way interaction) and dose response (number of modules used) effects at T3

	3-way interaction^a^		Number of modules used^b^				
Outcome measure	*Chi-Square*	*p* value	*b*	*95% CI*		*Chi-Square*	*p* value
Emotional symptoms	10.01	0.12	−0.11	(−0.20;	−0.03)	6.84	0.009
Total score	9.78	0.13	−0.58	(−0.78;	−0.39)	26.71	<0.001
Impact	2.66	0.85	−0.05	(−0.12;	0.01)	3.02	0.082
Conduct problems	10.95	0.09	−0.13	(−0.19;	−0.07)	16.17	<0.001
Hyperactivity/inattention	10.47	0.11	−0.25	(−0.32;	−0.18)	33.70	<0.001
Problems with peers	4.89	0.56	−0.10	(−0.18;	−0.02)	6.19	0.013
Prosocial behavior	11.71	0.07	0.11	(0.04;	0.18)	8.94	0.003

The unstandardized regression coefficient (*b*) and associated 95% CI come from Analysis of GEE Parameter Estimates, while the test statistics (chi-square and *p*-value) come from a Type 3 GEE Analysis. These tests are standard output from SAS proc genmod; model / type3.

^a^*N* = 866.

^b^*N*_intervention_ = 449.

### Dose-response effects in the intervention group

Approximately 80% of children and adolescents in the intervention group engaged with the SES NXT intervention; most youths (~72%) accessed the first module the same day as or the day after the T1 survey was completed and engaged with the modules over the course of 1 day (~93%). Tables depicting the frequency of the number of modules completed and the frequency of completion of specific modules can be found in the supplementary materials (Supplementary Tables 5 and 6). As shown in Table 4, a clear dose-response effect was observed where greater module engagement was significantly associated with lower levels of emotional symptoms, total psychological difficulties, conduct problems, hyperactivity/inattention, and peer problems, as well as higher prosocial behavior at T3. Augmenting this analysis, Supplementary Fig. 3 depicts the association between changes in emotional symptoms scores from T1 to T3 and the number of modules used, for each intervention age group. The figure shows that greater module use was associated with greater improvements in emotional symptoms from T1 to T3. The pattern was similar for all other outcomes (i.e., total psychological difficulties, impact score, conduct problems, hyperactivity/inattention, peer problems, and prosocial behavior).

As post hoc analyses, we followed up by testing whether higher engagement with the various intervention themes (see Table 6) was related to the outcomes at T3. Supplementary Table 7 presents these results. Of the four themes (family constellations, practical matters, emotional aspects of parental divorce, and agency), only emotional aspects of parental divorce showed a pattern of significance. Specifically, greater use of the modules within this theme was associated with lower levels of total psychological difficulties, impact of problems, conduct problems, hyperactivity/inattention, and peer problems at T3.

Intervention use was further contextualized by examining whether T1 sociodemographic factors (i.e., child gender and age, parent gender and age, parent education and income), child well-being (i.e., SDQ overall score and impact score), and parental mental health (i.e., depression and anxiety) predicted greater module use. Supplementary Table 8 demonstrates that for children and adolescents aged 6–17, greater total psychological problems and greater impact of problems at baseline, as well as greater parental income predicted greater module use, whereas older parent age was associated with lesser module use. Supplementary Table 9 indicates that only parental age was associated with intervention use for 3–5-year-olds, such that older parental age was associated with 20% higher odds of intervention use.

### National norm comparisons and user feedback

**Fig. 2:**
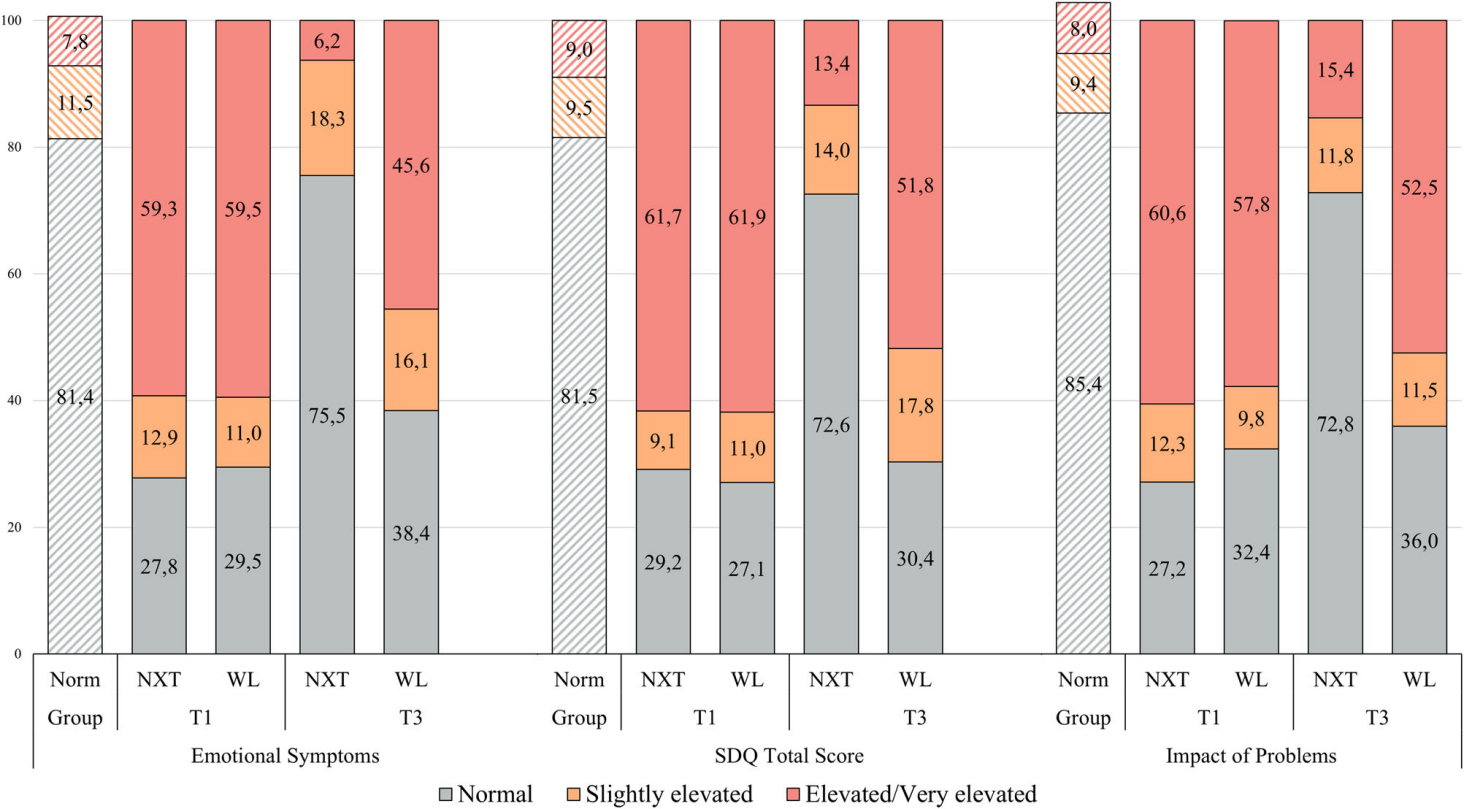
Distribution of “normal”, “slightly elevated”, and “elevated/very elevated” scores on the three primary outcomes, for intervention and WL group at T1 and T3. The norm group bars represent averages of the age- and gender-specific percentages provided in Arnfred et al.^45^. For impact of problems, these averages, when summed, equal 103% due to rounding (Arnfred et al.)^45^.

**Fig. 3:**
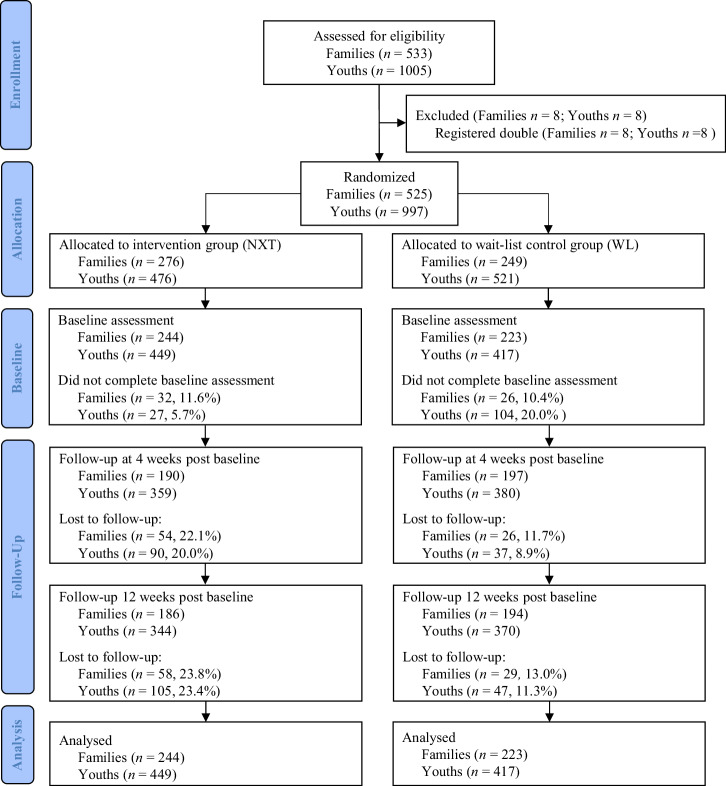
CONSORT diagram of participant flow through the trial. Lost-to-follow-up percentages at T2 and T3 are calculated with respect to the baseline sample sizes, as participants were sent the T3 survey, even if they did not complete the T2 survey.

To contextualize the findings, we categorized the children’s scores on the 3 primary outcomes (emotional symptoms, the SDQ total score, and the impact of problems score) at T1 and T3, into “normal”, “slightly elevated”, and “elevated/very elevated”, in accordance with the Danish age- and gender SDQ norms^45^. As can be seen from Fig. 3, over time, the intervention group’s scores approached or fell within normative ranges from T1 to T3, while the control group’s scores remained elevated from T1 to T3 (see Fig. 2). We also examined how many children and adolescents shifted from “elevated/very elevated” symptom levels at T1 to “normal” levels at T3. In the control group, approximately 10% made this shift (Emotional Symptoms: 12.23%; Total Difficulties: 8.15%; Impact Score: 8.39%), compared to about 47% in the intervention group (Emotional Symptoms: 46.55%; Total Difficulties: 48.33%; Impact Score: 47.44%).

Finally, the SDQ single-item follow-up question asking participants about self-perceived changes in problems since the start of the project indicated a significant, positive, and large user perceived effect. Specifically, we found that those in the intervention group reported greater improvements in problems since the start of the project (*M*_T2_ = 3.87, *SD*_T2_ = 0.1,18; *M*_T3_ = 4.17, *SD*_T3_ = 1.01) as compared to those in the WL control group (*M*_T2_ = 3.09, *SD*_T2_ = 0.89; *M*_T3_ = 3.18, *SD*_T3_ = 1.01) at both T2 (*M*_diff_ = -0.78, *z* = −11.05, *p* < 0.001, Cohen’s *d* = 0.74) and T3 (*M*_diff_ = −0.98, *z* = −13.38, *p* < 0.001, Cohen’s *d* = 0.97), with the magnitude of these differences being large in effect size. Combined, the study findings provide consistent support for both the primary and secondary study hypotheses 1–7.

## Discussion

This RCT offers robust evidence for the efficacy of the SES NXT digital intervention in improving mental health outcomes among children and adolescents who have experienced parental divorce. In a large and socio-demographically diverse Danish sample of 866 children and adolescents aged 3–17, the intervention led to medium to large and statistically significant reductions in emotional symptoms, total psychological difficulties, conduct problems, hyperactivity/inattention, peer relationship issues, and the impact of these problems in everyday life. In parallel, prosocial behavior improved significantly and substantially. These findings consistently support study hypotheses 1–7 and underscore SES NXT’s potential as an effective digital tool to support children and adolescents’ mental health across age groups and backgrounds following parental divorce. As outlined in the Introduction, prior interventions in the area have shown variable outcome results, limited scalability, and restricted age applicability. In contrast, SES NXT demonstrated robust, cross-age-group efficacy with larger effect sizes.

What distinguishes the present study results is not only the magnitude and breadth of the effects observed but the triangulation of findings from three independent sources: First, an absolute symptom reduction where, compared to the control group, intervention group participants showed reductions in mental health problems and improved well-being, with effect sizes ranging from moderate to large (Cohen’s (*d*) = 0.47–0.71), indicating meaningful changes across a wide array of psychological domains. Second, normative benchmarking where, for the intervention group, participants’ scores approached or fell within Danish national normative ranges for children and adolescents’ mental health and well-being at T3, while the control group’s score remained elevated, further validating the real-world relevance of the observed improvements. Third, user-perceived improvements where participants in the intervention group compared to controls reported large reductions in problems from study enrollment to study conclusion. This direct subjective validation aligns with the objective outcomes, enhancing the ecological validity of the SES NXT intervention^46^. Combined, these converging lines of evidence and triangulated pattern strengthen confidence in the intervention’s impact and practical utility in the everyday lives of youth who experience parental divorce^47^.

To understand why SES NXT produced substantial and wide-ranging effects, it may be instructive to consider both the developmental process and the theoretically grounded and practitioner-informed mechanisms^46–48^. SES NXT was developed over several years through an iterative, user-centered design process combining academic, clinical, and digital expertise. The intervention’s structure and content were informed by multidisciplinary collaboration among psychologists, family therapists, educators, and digital design specialists, alongside iterative input from children, parents, and practitioners. This co-design approach ensured that the material was developmentally attuned, linguistically accessible, and engaging across the full age range of children and adolescents.

Theoretically, the intervention is firmly situated within the DSAP, which conceptualizes children and adolescents’ adjustment to divorce as a gradual, multifactorial process shaped by exposure to stressors, individual psychological resilience, and the availability of supportive (protective) resources^18,25–27^. SES NXT operationalizes this model by serving as a readily available structural buffer/resource that delivers age-sensitive, targeted support at critical junctures of family transition. Rather than treating adjustment as a one-time crisis response, the intervention aligns with the DSAP’s view of adaptation as dynamic, cumulative, and shaped over time by adequately navigating evolving family arrangements such as living in two households or coping with ongoing parental conflict^15^. Further, its modular design allows children and adolescents to engage with the themes most applicable to them, including setting boundaries, navigating changing emotions, or dealing with parental conflict^15^. In this way, the contents of SES NXT have the potential to accompany children and adolescents through the evolving challenges and different needs of post-divorce life, aligning with the core rational of the DSAP^18,25–27^.

Practitioner experience and input were collected and incorporated throughout the intervention’s development^46–48^. Informed by established clinical and social practice, SES NXT draws on mechanisms frequently emphasized in face-to-face divorce interventions, particularly those used in group-based sessions with children and adolescents, such as promoting normalization through peer recognition and modeling^49^. By exposing children and adolescents to relatable narratives and modeled experiences, the intervention affirms their emotions and situational challenges as valid, thereby reducing isolation and strengthening a sense of belonging^50,51^. Informed by practice, SES NXT also encourages parental co-participation with children and younger youth, creating a shared point of reference that may facilitate mutual reflection and discussion within families. Moreover, informed by both literature and practitioner experience, the intervention actively promotes agency by guiding children and adolescents to explore tailored, interactive content that supports identification of needs, boundary setting, and problem solving within a flexible, self-directed framework^15^. Collectively, these features suggest that SES NXT introduces a constructive and well-calibrated disruption within the family system sufficient to catalyze new insights and interactional patterns without destabilizing existing bonds. Finally, the low threshold for engaging, easy accessibility, and the intervention’s self-directed nature also make it especially accessible to families who might otherwise be hesitant or unable to engage with formal support systems. This “dual capacity” to empower families on their own terms while fostering deeper psychological adjustment may also be central to the intervention’s observed efficacy.

However, while SES NXT is grounded in a co-design approach, informed by theory, and incorporates practitioner-derived mechanisms such as normalization, emotional literacy, and agency, the present trial did not include direct measures of these constructs. Consequently, we cannot empirically disentangle which mechanisms primarily accounted for the observed reductions in increased mental health and well-being. It is plausible that different mechanisms contributed across developmental stages and family contexts; for instance, gains in emotional literacy and self-efficacy may have played a greater role among older children and adolescents, whereas improved parental attunement and emotional scaffolding may have been more central among younger participants^49^. Given that family dynamics, digital engagement patterns, and individual coping capacities vary substantially across users, SES NXT likely operates through multiple, interacting pathways rather than a single, uniform mechanism. Future research should therefore examine these potential mediators directly, incorporating both child/adolescent self-report and parental reports, and testing models of change across developmental subgroups in both this and other similar digital interventions. Nevertheless, despite these current limitations, the consistency and magnitude of outcome improvements across age groups suggest that the intervention is effective in producing meaningful and substantial change in mental health and well-being among the target group.

Considering the study’s context, the observed effects of SES NXT may also have been helped by the structural and cultural conditions in Denmark, which are highly conducive to digital interventions. As a high-income country with comprehensive welfare and healthcare systems, Denmark has become a global leader in the digitalization of public services, promoting widespread digital access and literacy among both children and parents^52^. Denmark, therefore, provides a particularly supportive context for the implementation of scalable digital interventions, which may contribute to higher user acceptability and uptake of these solutions, as also reflected in the high average number of SES NXT modules used by participants in this study. Specifically, the intervention’s modular structure and cross-device accessibility align well with how Danish families typically engage with information, such as through digital interactions with public institutions. Additionally, SES NXT aligns well with national policy, which may have further reinforced its perceived relevance. For example, the intervention includes a module on children’s rights grounded in the UN Convention on the Rights of the Child, which has also shaped the 2024 Danish legislative reform *Barnets Lov* (“The Child’s Law”). This law requires that all decisions made by public authorities to prioritize the child’s best interests. Embedding a policy-relevant module based on international and national legal frameworks likely strengthened institutional support for the intervention which may also have increased the user-perceived relevance and credibility of the intervention. In fact, in this study, the children’s rights module was the most used module among the 13–17-year-olds and the second most used among the 9–12-year-olds (see Supplementary Table 6). These favorable conditions may not be present in all countries. Although SES NXT is easy to access and navigate, in countries with more limited digital literacy or lower levels of institutional support or a different cultural context, the use and effect of similar digital interventions could differ substantially. Future research should therefore examine whether SES NXT’s outcomes can be replicated in more varied sociocultural and technological contexts.

Another key strength of the study is that over 1000 children and adolescents from all regions of Denmark were recruited during the 16-month trial period, demonstrating the scalability of the SES NXT intervention. Notably, a substantial proportion of participants came from lower socioeconomic backgrounds, a group often underrepresented in traditional divorce and mental health support services^46^. This is particularly significant, as these families are less likely to access or benefit from conventional in-person interventions due to barriers such as stigma, financial and time constraints, or limited awareness^53,54^. The successful reach of SES NXT to this underserved population thus highlights its potential to reduce disparities in post-divorce support.

Despite this study’s strengths, including a large sample size, an RCT design, and the use of a validated and much used measure, central study limitations should be acknowledged. First, although the study employed a robust RCT design, intention-to-treat analyses, and achieved good retention rates at follow-up, there was notable drop-out from randomization to analysis. While this is a common challenge in RCTs, it is still worth noting when interpreting the results. Second, the follow-up was limited to 12 weeks, so it remains to be seen whether these gains are sustained longer term. Third, due to the reliance on self- and parent-reports, some self-report bias cannot be excluded. Triangulation with clinician ratings or school data in future work would therefore enhance the robustness of results. Fourth, the design did not allow for a detailed assessment of what participants learned or retained during intervention consumption, nor of engagement fidelity or intensity across participants, all of which could have influenced or moderated the outcomes. Fifth, although the study included a large sample from across Denmark, the sample was self-selected which limits the generalizability of results. Relatedly, contrary to typical practice, we incentivized use of the intervention and study participation which may impact conclusions about the generalizability of engagement and sustainability of the intervention. Lastly, the cohort included families who, on average, had been divorced for a substantial period (mean was 2 years). This may have made them especially receptive to support, potentially amplifying the observed intervention effects compared to groups with more recent divorces.

In conclusion, the SES NXT intervention represents a promising new digital avenue for supporting youth in divorced or blended families, suggesting that its use is associated with improved mental health and well-being of children of divorce.

## Methods

### Study design

The SES NXT trial was designed as a randomized controlled, parallel-group, superiority trial that compared the digital SES NXT intervention group with a wait-list (WL) control group. The primary endpoint was 12 weeks post baseline (T3). Figure 1 presents the CONSORT diagram.

### Ethical considerations

All procedures were in accordance with the ethical standards of the institutional and national research committee and with the 1964 Declaration of Helsinki and its later amendments or comparable ethical standards. We received ethical approval from the University of Copenhagen Research Ethics Committee for Science and Health (case number 504-0290/21-5000) and from the Danish Data Protections Agency (case number 514-0699/22-3000). The study was exempt from further ethical evaluations following the rules and regulations as set forth by the Scientific Ethical Committees of Denmark (i.e., national ethics approval was not required). The protocol was registered and posted on the 8th of March 2023 with clinicaltrials.gov (ClinicalTrials.gov Identifier: NCT05760820, https://clinicaltrials.gov/study/NCT05760820). We have created an Open Science Framework (OSF) project for the overall project (https://osf.io/wusjk/), which contains study-relevant documents including the measures document and codebook, with both English and Danish language versions of the items, power simulation code, the intervention white paper (detailing the intervention content), and additional methodological information. Manuscript-specific files can be located in a specific OSF project folder (https://osf.io/wyu92/) and include analysis code and output files. The procedure and measures are described in detail in Øverup et al.^15^. There were no deviations from the procedures described therein, with the exception of the missing data imputation strategy, which we changed to single imputation, as the amount of missing data was relatively low (roughly 12%). Additionally, a supplementary materials file accompanies this manuscript; this file contains additional results tables.

### Sample size

We simulated the data structure and expected distribution to determine the minimum sample size for this study using R, version 4.4.1. The sample size was calculated with reference to the primary hypothesis (H1), aiming for a power of 90% and an alpha level of 0.05. The estimate of the outcome pooled standard deviation was based on the post-test results from Pelleboer-Gunnick and colleagues’^55^ study of a school-based intervention for children of divorce, which also used the emotional symptoms subscale from the SDQ as a primary outcome. We powered the study to be able to detect a small to moderate effect, as evidenced by a raw mean difference of 0.40 in the SDQ emotional symptoms subscale score between the intervention and control group. The test of relevance is a cross-sectional multi-level model, with children and adolescents nested within parent. We used a generalized estimating equation (GEE), as these make fewer distributional assumptions and yield more robust standard errors. With these parameters, the simulation suggested that roughly 700 clusters (families consisting of one parent with their children) would be needed. On this basis, we decided to close study enrollment when the target sample was reached or on August 31st, 2024, whichever came first.

### Participants

To be eligible for study participation, parents had to be divorced, have at least one custodial child between the ages of 3 and 17 at study inclusion, access to an electronic device with internet (e.g., phone, tablet, or computer), and the ability to read and understand Danish, as all materials were provided in Danish only. Participants were recruited between May 1st, 2023, and August 31st, 2024, and data collection ended on January 31st, 2025. A total of 533 families and 1,005 children and adolescents were assessed for eligibility. A total of 66 families and 139 youth were excluded due to double registration or failure to provide baseline data, leaving 467 families and 866 youth in the intention-to-treat (ITT) analyzes of the RCT (see Fig. 3 for the CONSORT diagram).

Table 5 provides a breakdown of the demographic makeup of the sample. The average age of parent participants was 40.2 years. Close to half of the parent population had below-average income and low education. On average, the relationship lasted 10.9 years and, on average, the relationship ended 2.0 years prior to study enrollment. About half of the parent participants indicated that they were the divorce initiators, and a majority of parents participants indicated that either they and/or their partner had new partners. It was predominantly biological mothers who signed the family up for study participation, and the ex-partner was almost exclusively the child’s biological other parent. The most frequent number of children per family was two. The average age of youth participants was 9 years with a roughly equal gender distribution. The average number of modules engaged in the SES NXT intervention was 3.9.

**Table 5:** Baseline characteristics of participants in the full RCT ITT population

Characteristic		Study group^d^	
Demographic, Youth	NXT (*n* = 449)	WL (*n* = 417)	All (*N* = 866)
Girls	229 (51.0)	209 (50.1)	438 (50.6)
Age, mean (SD)	*9.0 (4.1)*	*9.0 (4.3)*	*9.0 (4.2)*
Survey age group			
3–5	109 (24.2)	108 (25.9)	217 (25.1)
6–10	193 (43.0)	162 (38.9)	355 (41.0)
11–17	147 (32.7)	147 (35.3)	294 (34.0)
Intervention age group			
3–5	109 (24.3)	108 (25.9)	217 (25.06)
6–8	115 (25.6)	109 (26.1)	224 (25.87)
9–12	122 (27.2)	96 (23.0)	218 (25.17)
13–17	103 (22.9)	104 (24.9)	207 (23.9)
Modules completed by intervention age group, mean (SD)	*4.0 (2.4)*		
3–5 ^a^	*1.00 (0.0)*	--	--
6–8	*4.82 (1.8)*	--	--
9–12	*5.30 (2.2)*	--	--
13–17	*5.00 (1.6)*	--	--
Demographic, Parent	NXT (*n* = 244)	WL (*n* = 223)	All (*N* = 467)
Age, mean (SD)	*40.2 (6.0)*	*40.3 (6.4)*	*40.2 (6.2)*
Parent registered as informant			
Biological mother	158 (64.8)	147 (65.9)	305 (65.3)
Biological father	86 (35.3)	76 (34.1)	162 (34.7)
Ex-partner, in relation to child			
Biological mother	85 (34.8)	72 (32.3)	157 (33.6)
Biological father	157 (64.3)	144 (64.6)	301 (64.5)
Bonus parent/other	2 (0.8)	7 (3.1)	9 (1.9)
Region, Parent			
Capital Region of Denmark	84 (34.4)	80 (35.9)	164 (35.1)
Region Zealand	65 (26.6)	63 (28.3)	128 (27.4)
Region of Southern Denmark	31 (12.7)	33 (14.8)	64 (13.7)
Central Denmark Region	46 (18.9)	30 (13.5)	76 (16.3)
North Denmark Region	18 (7.4)	17 (7.6)	35 (7.5)
Parent highest educational level			
Low	112 (45.9)	109 (48.9)	221 (47.3)
Medium	73 (29.9)	70 (31.4)	143 (30.6)
High	59 (24.2)	44 (19.7)	103 (22.1)
Parent monthly income in DKK^b^			
Below average (<10,000–40,000)	103 (42.2)	110 (49.3)	213 (45.6)
Average (40,001–50,000)	68 (27.9)	47 (21.1)	115 (24.6)
Above average (50,001- >80,000)	73 (30.0)	66 (29.6)	139 (29.7)
Relationship status^c^			
Divorced	133 (54.5)	111 (49.8)	244 (52.3)
Marital duration, mean (SD)	*11.0 (5.5)*	*10.8 (5.5)*	*10.9 (5.5)*
Time since divorce, mean (SD)	*1.9 (2.4)*	*2.1 (2.6)*	*2.0 (2.5)*
Split/break-up	94 (38.5)	97 (43.5)	191 (40.9)
Relationship duration, mean (SD)	*11.1 (4.6)*	*10.7 (4.6)*	*10.9 (4.6)*
Time since break-up, mean (SD)	*1.9 (1.8)*	*2.1 (3.0)*	*2.0 (2.5)*
Break-up initiator			
Predominantly me	120 (49.2)	110 (49.3)	230 (49.3)
Mutual	47 (19.3)	45 (20.2)	92 (19.7)
Predominantly ex-partner	77 (31.6)	68 (30.5)	145 (31.1)
New Partner			
Neither have new partners	96 (39.3)	91 (40.8)	187 (40.4)
Participant does, former spouse does not	55 (22.5)	49 (22.0)	104 (22.3)
Participant does not, former spouse does	48 (19.7)	51 (22.9)	99 (21.2)
Both have new partners	45 (18.4)	32 (14.4)	77 (16.5)
No. of children and adolescents signed up per family			
1	52 (21.3)	54 (24.2)	106 (22.7)
2	179 (73.4)	147 (65.9)	326 (69.8)
3	13 (5.3)	19 (8.5)	32 (6.8)
4	--	3 (1.4)	3 (0.6)

^a^The age group 3–5 year-olds accessed 4 themes, built as 1 module.

^b^The average monthly income in Denmark was 46.927 DKK in 2023.

^c^Some participants did not report their relationship status or provide dates for key events (e.g., start of relationship, marrying, separating, or divorcing), so percentages may not sum to 100 and sample sizes (*N*) vary. Missing data were not imputed. Time is shown in years.

^d^Unless otherwise indicated, data are expressed as number (percentage) of participants. Percentages have been rounded and may not total 100. Mean (SD) are presented in italics.

### Attrition bias

There was evidence of differential attrition from the study (see Fig. 3); specifically, 20.0% of youth participants in the intervention but only 8.9% of youth in the WL group did not complete the T2 measures. Drop-out increased for both the intervention group (23.4%) and the WL group (11.3%) at T3, though the magnitude of the attrition remained disparate, with less drop-out from the WL group than the intervention group.

We therefore examined whether attrition (i.e., responding to only T1 (1) vs. responding to 2 or more time points (0)) was predicted by baseline sociodemographic variables (i.e., child gender and age, parent gender and age, parent income and education), child well-being (i.e., SDQ overall score and impact of problems score), and parental mental health (i.e., depression and anxiety) using a logistic regression analysis. Supplementary Table 2 shows that four variables were significantly associated with attrition. Being in the WL group (*OR* = 0.43), experiencing a greater impact of problems (*OR* = 0.88), and having a higher parental income (*OR* = 0.81) were associated with lower odds of dropping out, whereas older parental age was associated with higher odds of dropping out (*OR* = 1.07).

### Randomization

Families were randomized to either the SES NXT intervention or a wait-list control (WL) group. Randomization was performed electronically upon sign-up, with a random number generator, and randomization occurred at the family level, such that parents and children and adolescents were randomized to the same group, at a 1:1 allocation ratio.

### Procedure

Families were recruited through the National Agency of Family Law and 21 Danish municipalities that are geographically spread throughout Denmark and socio-demographically diverse in population composition. Municipal officials distributed informational fliers through “AULA” or referred the families directly to an online sign-up form. AULA is the national digital communication platform used by schools and daycare institutions in Denmark. It facilitates secure and structured communication between parents, teachers, and school staff, and is widely used for sharing information about children’s education, activities, schedules, and school-related announcements.

One custodial parent completed the sign-up form, providing the name, age, and contact details for each child they wished to enroll. They also submitted their own email and/or phone number, or that of the children. Consent was obtained at the end of the sign-up form, with custodial parents providing consent on behalf of themselves and their children. Given the online nature of the study, child assent could not be collected directly; however, parents were encouraged to discuss participation and use of the SES NXT intervention with their children and assist them as needed.

Parents and adolescents aged 11–17 received an email and/or a text message with a direct link to the baseline (T1) survey. Parents completed a brief questionnaire about themselves and also responded on behalf of their children aged 3–10 at baseline (T1) and at both follow-up assessments (T2 and T3). Adolescents aged 11–17 at study enrollment completed the questionnaires themselves. If an adolescent lacked personal contact information, parents could receive and forward the survey link to them. Group allocation was revealed immediately upon completion of the T1 survey, after which participants in the intervention group were granted immediate unrestricted access to the SES NXT platform. User accounts were automatically generated at sign-up, and participants subsequently selected their own usernames and access codes to ensure security and anonymity.

Follow-up surveys were administered 4 weeks (T2) and 12 weeks (T3) from baseline (T1). The T3 follow-up survey was administered regardless of whether the T2 follow-up survey had been completed. Participants in the waitlist control group received access to SES NXT at study completion (90 days following the completion of T1) regardless of whether surveys at T2 or T3 had been completed.

To enhance retention and participation, two strategies were implemented: (1) Reminder messages were sent 2 and 5 days after each survey link was distributed to all study participants; and (2) participants could earn two cinema tickets (value: 180 DKK/24 EUR) per child. Participants in the WL group earned one ticket for completing two surveys and a second for completing all three surveys. Intervention group participants earned one ticket for completing two surveys and three intervention modules, and a second for completing all three surveys and three intervention modules. To increase participation and use of the intervention, we implemented two e-mail reminders for the intervention group: (1) first a reminder for the participants to create a user on the platform, and (2) second, a reminder to complete three modules to be eligible to receive the cinema ticket.

### Intervention

The SES NXT intervention is a brief, modular and age-adjusted intervention developed in line with the DSAP outlined above^25^. Each module comprises a theme identified in the literature as central to children and adolescents experiencing divorce (e.g., living in two homes, bonus families, and parental conflict). The intervention uses minimal text and relies heavily on interactive engaging contents and videos, motion graphics, and voice-overs. The modules vary in length of time; however, it is estimated that the average time per module is about 7 min. Thus, for age group 6–8, completion of all 8 modules is estimated to take about an hour, while for age groups 9–12 and 13–17, completion of all 10 modules is estimated to take about 1 h and 15 min.

The intervention is tailored and adaptive to four age groups corresponding to the Danish daycare and school system (i.e., ages 3–5, 6–8, 9–12 and 13–17 years) and addresses key factors associated with adjustment to parental divorce, such as perceived support^56^, coping skills^31^, and child-parent relations and communication^29,31,57,58^. Given that divorce is a heterogeneous process, and the experience of divorce is different for each individual, participants could freely choose the modules that were most relevant to them. The modules could be completed in any order, as little or much as desired, and repeated as needed. Table 6 provides an overview of themes in the SES NXT intervention by age. Parents were encouraged to support, supervise, and engage with the intervention alongside their children, with increasing emphasis on parental involvement the younger the participant. Please see the study protocol for more details regarding the intervention^15^ as well as Supplementary Fig. 1, which details the expected parent involvement. Of note, we were unable to collect information about parent involvement in module completion. The interventions for the different age groups were designed in ways that younger children were encouraged more than older children and adolescents to seek out their parents for help.

**Table 6:** Intervention content overview, by age group in the intervention

Theme	3–5 years	6–8 years	9–12 years	13–17 years
Family constellations				
My family	X			
The bonus family		X	X	X
Practical matters				
Changeover day	X			
Living in two places		X	X	X
Packing your bag		X	X	X
Emotional aspects of parental divorce				
Missing someone means you care	X			
Feelings	X	X		
Understand your feelings			X	X
When it has just happened		X	X	X
Tell your story			X	X
Agency				
Find an important adult		X	X	X
Learn to say yes and no		X	X	X
My parents are not getting along		X	X	X
My rights			X	X

The intervention for age 3–5 years consisted of 1 module that contained the four themes indicated in the column above.

### Measures

In terms of demographic variables, parents reported their *gender* (0 = male, 1 = female) and *age* (in years). *Educational level* was categorized into three groups: low (e.g., primary school, vocational education), medium (e.g., medium-cycle tertiary education, bachelor’s degree), and high (e.g., master’s degree or higher). *Monthly income* was recorded using a nine-point scale in 10,000 DKK increments (approximately 1500 USD), ranging from below 10,000 DKK to above 80,000 DKK. Parents also provided information on their *past* *and current relationship status*, including whether they were legally divorced or had split/broken up if not legally married, the duration of this relationship, time since divorce, who initiated the break-up (i.e., “more me,” “mutual,” or “more the other”), and whether they or their former partner had entered a new relationship. Parents reported on the *gender of their child* aged 3–10, while adolescents aged 11–17 self-reported their gender (0 = male, 1 = female). *Child age* was derived from the birth date provided at sign-up.

*Parental mental health* was measured using the PHQ-2^59^ and GAD-2^60^, respectively. Each scale contains two items rated on a 4-point scale: 0 = “Not at all,” 1 = “Several days,” 2 = “More than half the days,” and 3 = “Nearly every day,” assessing symptom frequency of depression (PHQ; *α* = 0.70/0.51/0.60, for each time point) and generalized anxiety (GAD; *α* = 0.78/0.64/0.75, for each time point) over the previous 2 weeks. Higher scores denote worse mental health. Scores on the PHQ-2 and GAD-2 were included as covariates in the analyzes.

*Intervention use* was automatically tracked by the SES NXT platform whenever a module was started or completed. A composite variable was constructed to reflect the total number of modules started or completed by each participant. This measure was used in secondary analyzes to examine potential dose-response effects.

For the *outcomes measures* of interest, we used the Strengths and Difficulties Questionnaire (SDQ)^61,62^ to assess the children and adolescents’ emotional, behavioral, and social functioning. Parents completed a proxy version for children aged 3–10, while adolescents aged 11–17 completed a self-report version. The SDQ consists of 25 items rated on a 3-point scale (0 = “Not true,” 1 = “Somewhat true,” 2 = “Certainly true”), generating five subscales: Emotional Symptoms, Conduct Problems, Hyperactivity/Inattention, Peer Relationship Problems, and Prosocial Behavior (each ranging from 0 to 10) with higher scores indicating more symptom severity or higher prosocial behavior. The Total Difficulties Score is calculated by summing the four problem subscales (range: 0–40), with higher scores indicating greater psychological difficulties (i.e., prosocial behavior is not included in the total difficulty score). The extended version of the SDQ includes an additional five-item Impact Scale measuring the extent to which the child’s difficulties interfere with daily life (e.g., at home, with friends, at school), rated on a 4-point scale (0 = “Not at all”, 0 = “Only a little”, 1 = “A medium amount”, 2 = “A great deal”), with total scores ranging from 0 to 10. In addition, the extended version of the SDQ used included a single item evaluating the perceived change in the youth’s problems since study enrollment measured on a 6-point scale (1 = “much worse”, 2 = “a bit worse”, 3 = “about the same”, 4 = “a bit better”, 5 = “much better”, and 6 = “did not have a problem at the start pf the project”). Responses indicating no initial problem were recoded as missing (*N*_T2_ = 49; *N*_T3_ = 55). The SDQ has demonstrated good psychometric properties in Danish samples^45,63^. Primary outcomes were the SDQ Emotional Symptoms subscale, Total Difficulties Score, and Impact Scale (Hypotheses 1–3). Secondary outcomes included Conduct Problems (H4), Hyperactivity/Inattention (H5), Peer Relationship Problems (H6), and Prosocial Behavior (H7). Perceived change in the youth’s problems since study enrollment was included as an auxiliary outcome.

### Statistical analyses

To examine the effectiveness of the intervention, multilevel regressions were conducted to examine group differences on the primary outcomes: SDQ-Emotional (H1), SDQ-Total (H2), and SDQ-Impact (H3) at T3. These analyses were based on the intention-to-treat principle^64^. Single imputation using chained equations as implemented in the *R* package mice were applied to handle missing data; we imputed all variables, except relationship status and dates related to the relationship (e.g., when did the relationship start, when did the relationship end) for parent participants. The total amount of missing data was roughly 12%. Analyses were subsequently conducted in SAS 9.4, using the *proc genmod* procedure. The regression models were specified to allow for interdependence due to nesting of children within parent participants, using a GEE. Thus, in these analyses, child participant responses are the unit of analysis. Group membership (i.e., SES NXT intervention group (1) vs. wait-list control group (0)) was entered as a categorical predictor. Following examination of the primary outcomes, the same analyses were conducted with respect to the secondary outcomes (H4-7) and the SDQ item reflecting (self-) perceived changes in problems since study enrollment.

Additional follow-up analyses were executed to examine whether there was a differential change over time for the two groups, as reflected in a group-by-time interaction effect, with these analyses accounting for nesting of time within child. This was supplemented by an examination of a three-way interaction between group, time, and intervention age group (age groups 3–5, 6–8, 9–12, and 13–17 years), to examine whether the intervention was differentially effective for the different age groups. A significant interactive effect was followed up with pairwise comparisons between the intervention and WL group for each age group across each time point using a Bonferroni adjustment (adjusted *p*-value threshold = 0.05/12 comparisons per outcome = 0.004).

We also examined whether, for the intervention group, there were differences in the outcomes at T3 based on number of modules engaged with in the intervention (i.e., a dose-response effect), and engagement with the specific themes of the intervention; intervention age group was included in this analysis.

For all analyses, we included potential covariates. These were participants’ gender and age, participants’ baseline score on the outcome (this variable was not included in the group×time analyzes, as it was part of the outcome variable), and parental gender and age, educational level, income, and mental health symptoms. Moreover, a *p*-value of less than 0.05 was used as the threshold for statistical significance, since this value was used as the acceptable risk of type I error in our sample size estimation.

## Supplementary information

Supplementary Information

## Data Availability

The datasets generated and/or analyzed during the current study are not publicly available due to privacy concerns, but are available upon reasonable request from Gert Martin Hald, ghald@sund.ku.dk.

## Code availability

Manuscript-specific files can be located in a specific OSF project folder (https://osf.io/wyu92/) and include analysis code and output files, as generated in SAS, version 9.4.
