# Supplementary material for: Effects of the SES NXT intervention on mental health and well-being for children of divorce
Source: NPJ Digit Med. 2026 Apr 21;9:478. doi: 10.1038/s41746-026-02638-x (PMC13284408; doi:10.1038/s41746-026-02638-x)
Supplement: Supplementary file 1 — Supplementary Information [file 41746_2026_2638_MOESM1_ESM.pdf]

Supplementary materials for the manuscript

Effects of the SES NXT Intervention on Mental Health and Well-Being for Children of Divorce

Gert Martin Hald[1],

Daniel Bach Johnsen[1],

Theis Lange[1],

Andreas Nielsen Hald[1,2],

Søren Sander[1],

Camilla Stine Øverup[1]

[1] Department of Public Health, University of Copenhagen,

Øster Farimagsgade 5B, 1014 København K, Denmark.

[2]Department of Public Health, Aarhus University,

Bartholins Allé 2, 8000 Aarhus C, Denmark.

This file contains additional information and results tables that may be of interest to readers.

We have created an Open Science Framework project that contains information about the overall RCT: https://osf.io/wusjk

> This page contains item-level code books, power simulation code, the intervention white paper, and additional methodological information.

We have also created a manuscript-specific OSF page: https://osf.io/wyu92

> This page contains the analytic code and results output for this manuscript.

Intervention Description (from Øverup et al., 2025)

SESNXT is a digital intervention for children and adolescents aged 3-17 years, who experience parental relationship dissolution. The intervention includes various elements such as text, videos, motion graphics, and voice-overs, and highly interactional content such as digital activities and exercises. SESNXT is designed to be adaptive to the children and adolescents' age. The four age categories correspond to the Danish daycare and school system, with 3–5-year-olds attending daycare and kindergarten, ages 6-8 years representing pre-school and start of primary education, ages 9-12 years representing primary education and start of lower secondary education, and ages 13-17 years representing lower secondary education and start of secondary education (e.g., high school). The format of the intervention was adapted to reflect the cognitive, social, emotional, and digital competencies of each of those age groups. Specifically, the intervention for children aged 3-5 consists of videos with speak that the parent and child can watch together and contain dialogue questions for the parent to initiate conversations with the child about the theme in question. Children ages 6-8 can manage a digital tool on their own (with less parental guidance) but may not be able to read; therefore, the intervention consists of videos with speak and some short texts at a low reading level. For children and adolescents aged 9-12 and 13-17, the intervention consists of videos, texts, and interactive content, reflecting their greater reading level and digital competencies. However, the interventions for the two age groups differ with respect to their level of abstraction. Moreover, videos that contain other children (i.e., "peers") present children in the same age range as the participants (e.g., 9-12-year-olds watch videos of other 9-12-year-olds).

The intervention addresses key factors associated with adjustment to parental relationship dissolution, such as perceived support (Sorek, 2020), coping skills (O'Hara et al., 2019; Sandler et al., 2000), child-parent relations and communication (Gunter Castleton,

2020; Kunz, 2001; O'Hara et al., 2019; Wal et al., 2021), and the interpretation and meaning made of the relationship dissolution (e.g., loss, gain, change, healing) (Brand et al., 2017; Venta &Walker, 2021). Table 2 provides an overview of the modules included in the intervention for each age group. Each module comprises a theme identified in the literature as central to children and adolescents experiencing parental relationship dissolution (e.g., living in two homes, bonus families, and parental conflict). Most of the content in the intervention is covered across the age groups, though in age-appropriate ways. An example of this is the module(s) concerning emotions (i.e., "Feelings" for 3–5-year-olds and 6–8-year-olds and "Understand your feelings" for 9–12-year-olds and 13–17-year-olds), which covers the various emotions that one might experience in response to a parental relationship dissolution. Different emotions are described: sadness and longing, guilt, joy, fear and insecurity, anger. For the two older age groups, the text for sadness and longing is: "Sadness and longing are the best! Because it shows you that someone or something means a lot to you. In other words, it's a sign of love. But... isn't it bad to be sad? No, actually not. It can be tough and hard, but if you were never sad, it would actually be a little sad." The texts are (often) the same for the 9-12- and 13–17-year-olds and they are introduced to an understanding of emotions as good, even though they can be difficult to experience.

Building on the example, the theme of longing is also addressed for the 3-8-year-olds. Here, the text is: "Everyone sometimes misses someone they care about. Both children and adults. To miss means thinking about someone who isn't there. Someone you care about. Maybe you can feel it in your stomach? Maybe you become a little sad? It could also be that you're looking forward to seeing the person you miss. That's totally okay." Children in the 3-5 and 6-8 age groups are introduced to the concept of "longing", a common emotional response, and are made to understand that they can feel this way when their parents have separated. In essence, for these modules, the intervention seeks to help the younger children

to mentalize various feelings, while the older children and adolescents are challenged to understand emotions at a higher level of abstraction, as they must recognize that what feels hard may also be important.

The guiding principles interwoven into the digital modules are: 1) The mind is social (Tomm, 1984): Human behavior, emotions, and cognitions are situated in a social context. 2) Re-connection (Perry, 2013): When children experience significant life changes, connections with existing values, hopes, or dreams may change. The child is guided to re-connect with these in the same or in an altered form, depending on new life circumstances. 3) Agency (White, 2008): The experience of and belief in the ability to influence the world around oneself and not only be the passive recipient of what happens around oneself. 4) Appropriate disturbance (Schjødt & Egeland, 2008): An 'appropriate disturbance' typically refers to a disruption or challenge that is constructive and suitable within a specific context. This type of disturbance is designed to evoke thought, encourage growth, or create necessary change without causing harm or undue stress. 5) Normalization (White, 2008): When children and adolescents face parental relationship dissolution, they may feel they have failed or done something wrong or think they are alone in these difficult emotions. Normalizing these feelings by showing them they are not alone can make them less distressing and intrusive. 6) Mentalization (Aasen & Fonagy, 2021): The ability to understand mental states of oneself and other.

The intervention can be accessed at any time during the trial by logging into the intervention website from a mobile smartphone, tablet, or computer. Given that divorce is a heterogeneous process, and the experience of divorce is different for each individual, participants choose the modules that are most relevant to them. The modules can be completed in any order, as little or much as desired, and repeated as needed.

Supplementary Figure 1. Demonstrating the Expected Level of Parent Involvement for each of the Intervention Age Groups.

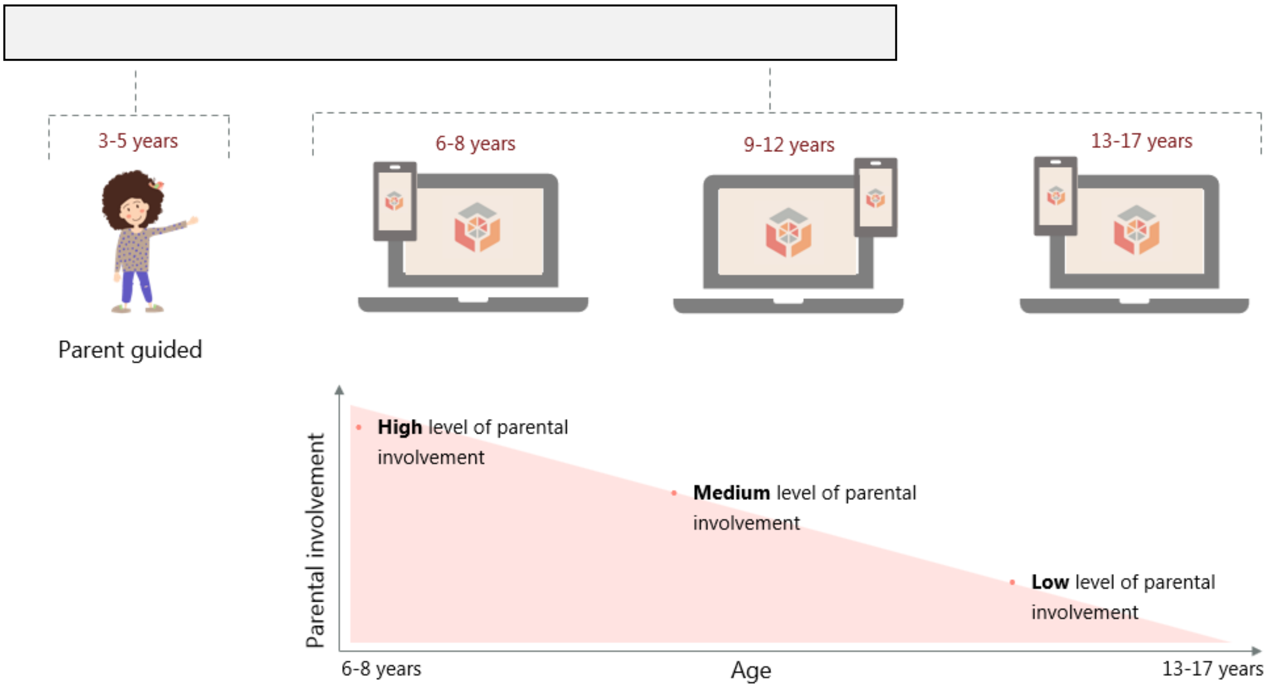

Supplementary Table 1 provides an overview of all measurements, including who completed the measurement (parent- vs. self-report) and for which ages.

Supplementary Table 2 provides the results of attrition bias analyses, in which we used baseline sociodemographic variables (child age and gender, parent age and gender, and parental education and income), child well-being (SDQ total and SDQ impact of problems), and parental mental health (depression and anxiety) to predict attrition (i.e., completing only 1 wave of measurement vs. completing 2 or more waves of measurements).

Supplementary Table 3 provides comparisons between intervention and WL group at T3, as derived from unadjusted analyses.

Supplementary Table 4 provides comparisons between intervention and WL group for each age group over time. **Supplementary Figure 2** demonstrates these differences visually.

Together, **Supplementary Table 4** and **Supplementary Figure 2** indicate that there were no significant differences at T1 between the intervention and WL group for all age groups, while at T2 and T3 there were significant differences between the intervention and WL for all age group – this pattern was evident across the majority of outcomes (for Hyperactivity/Inattention and Prosocial Behavior, three comparisons were non-significant at the Bonferroni-adjusted threshold).

Supplementary Table 5 displays the frequencies of engagement with number of modules, for the overall sample and well as for each intervention age group.

Supplementary Table 6 displays the frequencies of engagement with specific modules, for the three older intervention age groups (6-17 years of age).

Supplementary Table 7 displays the results of a regression that examines the association between the four intervention themes and T3 outcomes. Possible ranges (i.e., number of modules within each theme) can be found in Table 2 within the manuscript.

Supplementary figure 3 displays the association between number of modules and change in emotional symptoms from T1 to T3. Negative numbers indicate a lager improvement in symptoms from T1 to T3 (i.e., difference = T3 scores – T1 scores). Please see the manuscript-specific OSF page (https://osf.io/wyu92) for a PDF of figures for all outcomes.

Supplementary Table 8 displays the results of predicting number of modules used (i.e., intervention engagement) from baseline sociodemographic variables and baseline child overall well-being and impact of problems, and parent mental health. This table encompasses the results for 6-17-year-olds.

Supplementary Table 9 displays the results of predicting intervention engagement (i.e., yes = 1 vs. no = 0) from baseline sociodemographic variables and baseline child overall well-being and impact of problems, and parent mental health. This table encompasses the results for 3-5-year-olds.

Supplementary Table 1. ***Overview of Measurements***

	Parent Self-report	Age 3 Parent report	Age 4-10 Parent report	Age 11-17 Self-report
Socio-demographic questions and relationship/divorce questions	T1			
Divorce conflict (Hald, Strizzi et al., 2020)	T1, T2, T3			
Depression symptoms (PHQ-2; Kroenke et al., 2003)	T1, T2, T3			
Anxiety symptoms (GAD-2; Kroenke et al., 2007)	T1, T2, T3			
Strength and Difficulties Questionnaire (Goodman et al., 1998)		T1, T2, T3	T1, T2, T3	T1, T2, T3
Physical health: Somatization (SDQ, item 3), body dissatisfaction, sleep quality		T1, T2, T3	T1, T2, T3	T1, T2, T3
School and daycare attendance (Heyne et al., 2020)		T1, T2, T3	T1, T2, T3	T1, T2, T3
The Quality of My Life Questionnaire (Gong et al., 2007)				T1, T2, T3
Perceptions of parental conflict (Hald, Strizzi et al., 2020)				T1, T2, T3
Platform focus: Communicating needs, setting boundaries, and connecting with adults			T1, T2, T3 (age 6-10)	T1, T2, T3
Support services		T1	T1	T1

Note. T1 = baseline, T2 = follow-up at 4 weeks post-baseline, T3 = follow-up at 12 weeks post-baseline. The primary and secondary hypotheses specify the child-related questions as outcomes; parent self-report is used as covariates.

Supplementary Table 2. ***Examination of Potential Attrition Bias: Baseline Sociodemographic Variables, Child Well-being, and Parental Mental Health Predicting Attrition (i.e., completing only the T1 survey)***

Source	*b*	Chi-Square	*p*-value	*OR*
Group	**-0.866**	**9.45**	**0.002**	**.426**
Intervention age group	--	2.72	0.436	--
SDQ total score	-0.017	1.91	0.167	0.983
SDQ impact of problems	**-0.132**	**12.29**	**<.001**	**0.876**
Child gender	0.003	0.00	0.975	1.003
Child age	0.063	1.17	0.280	1.065
Parent gender	-0.342	1.08	0.299	0.710
Parent age	**0.068**	**6.95**	**0.008**	**1.070**
Parent income	**-0.212**	**4.93**	**0.026**	**0.809**
Parent educational level	--	0.56	0.757	--
Parent depression symptoms	0.099	0.75	0.387	1.104
Parent anxiety symptoms	0.040	0.15	0.696	1.041

Note. $N = 866$. The unstandardized regression coefficient (*b*) comes from Analysis of GEE Parameter Estimates, while the test statistics (chi-square and p-value) come from a Type 3 GEE Analysis. These tests are standard output from SAS proc genmod; model / type3 dist = binomial (to obtain results for a logistic regression). The unstandardized regression coefficients are only provided for continuous and binary predictors. For Gender-variables, female is the reference group. For the Group variable, intervention group was the reference group. Bold font indicates statistical significance. Analyses were conducted on the imputed data set.

Supplementary Table 3. ***Test of Differences between Intervention and WL Group at T3, Unadjusted***

	Between-group difference at T3				
**Outcome Measure**	**Mean diff.**	**(95% CI)**	***z* value**	***p* value**	**Cohen's *d***
Emotional symptoms	2.54	(2.11; 2.97)	11.61	<.001	.81
Total Score	6.86	(5.80; 7.91)	12.72	<.001	.88
Impact	2.14	(1.78; 2.50)	11.74	<.001	.82
Conduct problems	1.45	(1.16; 1.73)	9.89	<.001	.68
Hyperactivity/inattention	1.48	(1.18; 1.78)	9.66	<.001	.66
Problems with peers	1.38	(1.10; 1.67)	9.41	<.001	.65
Prosocial behavior	-1.31	(-1.63; -1.00)	-8.18	<.001	.56

Note. $N_{intervention} = 449$, $N_{WL} = 417$. T3 = 12-week follow-up. The results for the between-group difference come from Analysis of GEE Parameter Estimates (WL vs. intervention group), while the Group*time effect comes from a Type 3 GEE Analysis. These tests are standard output from SAS proc genmod; model / type3.

Supplementary Table 4. ***Comparisons between Intervention and WL Group, for each age group, across the three waves***

				Emotional Symptoms		Total Score		Impact Score		Conduct problems	
Group 1	Group 2	Wave	Age-group	Mean diff.	*p*-value	Mean diff.	*p*-value	Mean diff.	*p*-value	Mean diff.	*p*-value
WL	Intervention	T1	13-17 yr	-0.47	0.275	-0.56	0.578	-0.18	0.670	-0.19	0.529
WL	Intervention	T1	3-5 yr	0.17	0.668	-0.63	0.496	-0.31	0.411	-0.12	0.690
WL	Intervention	T1	6-8 yr	0.67	0.094	1.61	0.085	0.26	0.472	0.55	0.035
WL	Intervention	T1	9-12 yr	-0.57	0.158	-0.18	0.850	-0.19	0.614	0.05	0.850
WL	Intervention	T2	13-17 yr	**2.68**	**<.001**	**6.69**	**<.001**	**2.16**	**<.001**	**1.24**	**<.001**
WL	Intervention	T2	3-5 yr	**2.05**	**<.001**	**5.52**	**<.001**	**1.80**	**<.001**	**1.51**	**<.001**
WL	Intervention	T2	6-8 yr	**2.25**	**<.001**	**4.77**	**<.001**	**1.88**	**<.001**	**0.95**	**<.001**
WL	Intervention	T2	9-12 yr	**1.71**	**<.001**	**5.17**	**<.001**	**1.83**	**<.001**	**1.16**	**<.001**
WL	Intervention	T3	13-17 yr	**2.53**	**<.001**	**6.56**	**<.001**	**2.09**	**<.001**	**1.27**	**<.001**
WL	Intervention	T3	3-5 yr	**2.46**	**<.001**	**6.33**	**<.001**	**1.86**	**<.001**	**1.51**	**<.001**
WL	Intervention	T3	6-8 yr	**2.37**	**<.001**	**5.43**	**<.001**	**1.86**	**<.001**	**0.95**	**<.001**
WL	Intervention	T3	9-12 yr	**1.59**	**<.001**	**5.19**	**<.001**	**1.59**	**<.001**	**1.13**	**<.001**

Note. Bolded font indicates that the comparison is statistically significant at the Bonferroni-adjusted *p*-value threshold, $p = .004$. Covariates (i.e., participants' gender and age, parental gender and age, educational level, income, and parental time-varying mental health symptoms) were included in the analysis. T1 = baseline, T2 = 4-week follow-up, T3 = 12-week follow-up.

Supplementary Table 4 (continued). ***Comparisons between Intervention and WL group, for each age group, across the three waves***

				Hyperactivity/ inattention		Problems with peers		Prosocial Behavior	
Group 1	Group 2	Wave	Age-group	Mean diff.	*p*-value	Mean diff.	*p*-value	Mean diff.	*p*-value
WL	Intervention	T1	13-17 yr	-0.05	0.865	0.13	0.631	-0.07	0.776
WL	Intervention	T1	3-5 yr	-0.46	0.116	-0.23	0.378	0.44	0.148
WL	Intervention	T1	6-8 yr	0.05	0.857	0.33	0.217	-0.27	0.327
WL	Intervention	T1	9-12 yr	0.02	0.948	0.31	0.289	-0.32	0.269
WL	Intervention	T2	13-17 yr	**1.37**	**<.001**	**1.39**	**<.001**	**-1.35**	**<.001**
WL	Intervention	T2	3-5 yr	0.70	0.007	**1.25**	**<.001**	**-0.78**	**0.003**
WL	Intervention	T2	6-8 yr	0.51	0.065	**1.07**	**<.001**	**-0.96**	**0.001**
WL	Intervention	T2	9-12 yr	**1.14**	**<.001**	**1.16**	**<.001**	-0.66	0.021
WL	Intervention	T3	13-17 yr	**1.44**	**<.001**	**1.30**	**<.001**	**-1.51**	**<.001**
WL	Intervention	T3	3-5 yr	**1.31**	**<.001**	**1.04**	**<.001**	**-0.94**	**<.001**
WL	Intervention	T3	6-8 yr	**1.08**	**<.001**	**1.03**	**<.001**	**-0.90**	**<.001**
WL	Intervention	T3	9-12 yr	**1.00**	**<.001**	**1.46**	**<.001**	**-1.42**	**<.001**

Note. Bolded font indicates that the comparison is statistically significant at the Bonferroni-adjusted *p*-value threshold, $p = .004$. Covariates (i.e., participants' gender and age, parental gender and age, educational level, income, and parental time-varying mental health symptoms) were included in the analysis. T1 = baseline, T2 = 4-week follow-up, T3 = 12-week follow-up.

Figure 2. **Change in Scores over Time for Intervention and WL Group for each Intervention Age group.**

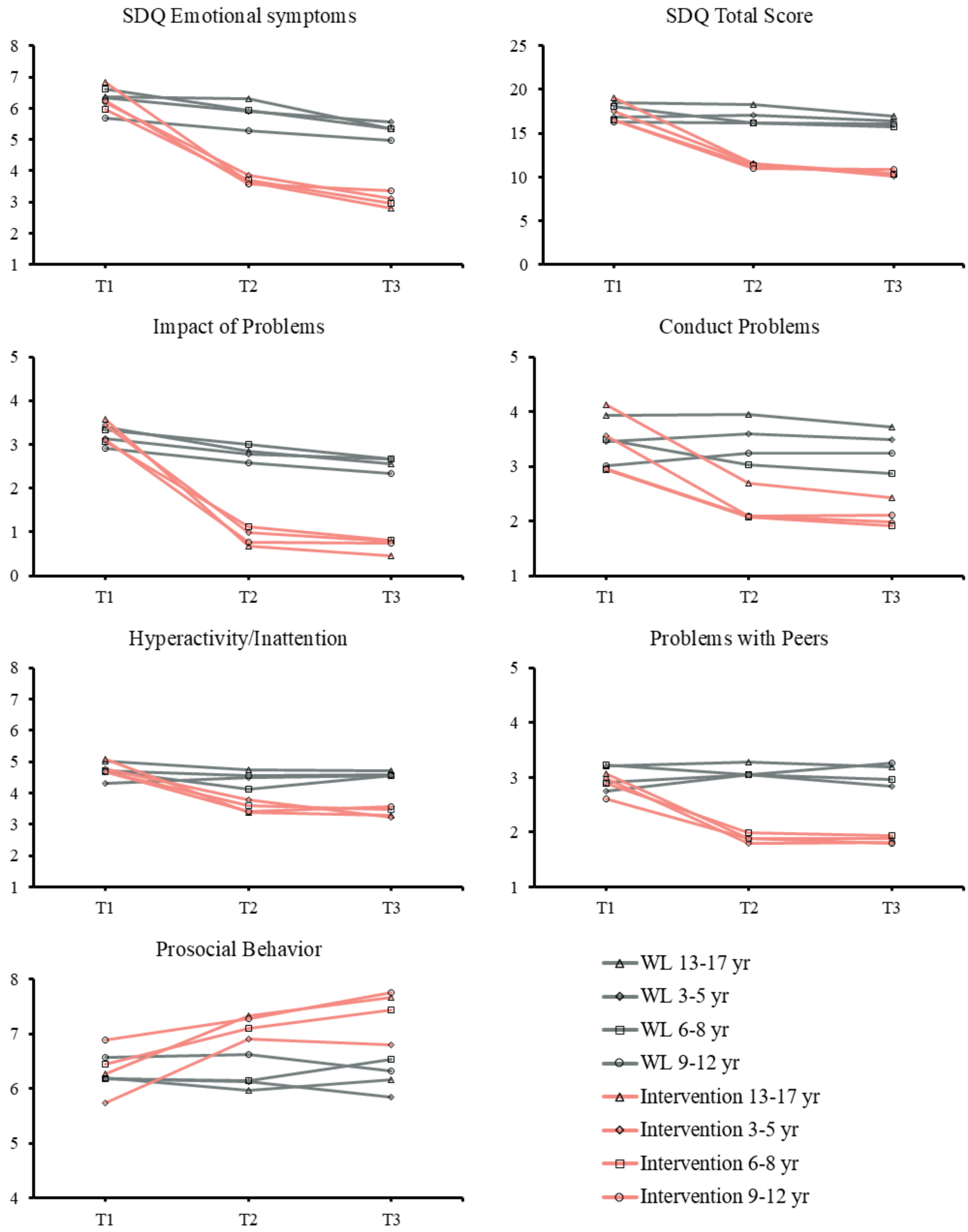

Supplementary Table 5. ***Frequencies of Number of Modules Completed***

	Overall (N = 449)		Intervention age group 3-5 (N = 109)		Intervention age group 6-8 (N = 115)		Intervention age group 9-12 (N = 122)		Intervention age group 13-17 (N = 103)	
Modules	Frequency	Percent	Frequency	Percent	Frequency	Percent	Frequency	Percent	Frequency	Percent
0	94	20.94	16	14.68	30	26.09	33	27.05	15	14.56
1	107	23.83	93	85.32	6	5.22	6	4.92	2	1.94
2	10	2.23	.	.	3	2.61	4	3.28	3	2.91
3	22	4.90	.	.	7	6.09	9	7.38	6	5.83
4	51	11.36	.	.	17	14.78	12	9.84	22	21.36
5	61	13.59	.	.	21	18.26	14	11.48	26	25.24
6	48	10.69	.	.	19	16.52	15	12.30	14	13.59
7	32	7.13	.	.	6	5.22	15	12.30	11	10.68
8	16	3.56	.	.	6	5.22	8	6.56	2	1.94
9	7	1.56	.	.	.	.	6	4.92	1	0.97
10	1	0.22	.	.	.	.	.	.	1	0.97

Note. For Intervention age group 3-5, the intervention consisted of 1 module that had four themes; for Intervention age group 6-8, the intervention consisted of 8 modules; for Intervention age group 9-12 and Intervention age group 13-17, the intervention consisted of 10 modules.

Supplementary Table 6. ***Displaying the Frequencies of Completion of the Modules***

**Theme**	Intervention age group 6-8 (N = 115)		Intervention age group 9-12 (N = 122)		Intervention age group 13-17 (N = 103)	
	Frequency	Percent	Frequency	Percent	Frequency	Percent
**Family Constellations**						
The Bonus Family	25	21.74	43	35.25	22	21.36
**Practical Matters**						
Living in Two Places	56	48.70	48	39.34	47	45.63
Packing Your Bag	37	32.17	32	26.23	18	17.48
**Emotional Aspects of Parental Divorce**						
Understand Your Feelings	74	64.35	55	45.08	51	49.51
When It has Just Happened	23	20.00	26	21.31	17	16.50
Tell Your Story	.	.	39	31.97	44	42.72
**Agency**						
Find an Important Adult	60	52.17	46	37.70	47	46.60
Learn To Say Yes and No	63	54.78	62	50.82	61	60.19
My Parents Are Not Getting Along	71	61.74	45	36.89	63	61.17
My Rights	.	.	56	45.90	68	66.02

Note. Frequencies are not displayed for age group 3-5, as they completed a single module that had four themes.

Supplementary Table 7. ***Depicting the Effect of Intervention Themes at 12-week Follow-up (T3) in the Intervention Group***

	Theme: Family Constellations			Theme: Practical Matters			Theme: Emotional Aspects of Parental Divorce			Theme: Agency		
**Outcome Measure**	***b* value**	***z* value**	***p* value**	***b* value**	***z* value**	***p* value**	***b* value**	***z* value**	***p* value**	***b* value**	***z* value**	***p* value**
Emotional symptoms	0.06	0.31	0.758	0.06	0.60	0.547	-0.13	-1.21	0.226	**-0.16**	**-2.58**	**0.010**
Total Score	0.16	0.29	0.774	-0.36	-1.30	0.193	**-1.22**	**-4.20**	**<.001**	-0.32	-1.92	0.055
Impact	-0.00	-0.01	0.994	-0.01	-0.19	0.848	**-0.19**	**-2.06**	**0.039**	0.00	0.04	0.968
Conduct problems	-0.06	-0.37	0.711	-0.13	-1.30	0.192	**-0.22**	**-2.34**	**0.020**	-0.08	-1.43	0.154
Hyperactivity/inattention	0.18	0.89	0.372	-0.17	-1.39	0.163	**-0.68**	**-5.54**	**<.001**	-0.06	-0.82	0.412
Problems with peers	0.05	0.25	0.806	-0.10	-0.83	0.409	**-0.33**	**-2.71**	**0.007**	0.02	0.28	0.776
Prosocial behavior	**0.60**	**3.11**	**0.002**	**0.32**	**2.63**	**0.009**	-0.01	-0.11	0.910	0.02	0.31	0.756

Note. $N_{intervention} = 449$. T1 = Baseline, T3 = 12-week follow-up. The results (unstandardized estimate *b*, *z*-value and *p*-value) come from Analysis of GEE Parameter Estimates (WL vs. intervention group). These tests are standard output from SAS proc genmod; model / type3. Bold font denotes statistical significance. Covariates (i.e., participants T1 score on the outcome, participants' gender and age, parental gender and age, educational level, income, and parental mental health symptoms at T3) were included in the analysis.

Supplementary Figure 3. Figure Demonstrating Changes in Emotional Symptoms by Number of Modules Used across Age Groups.

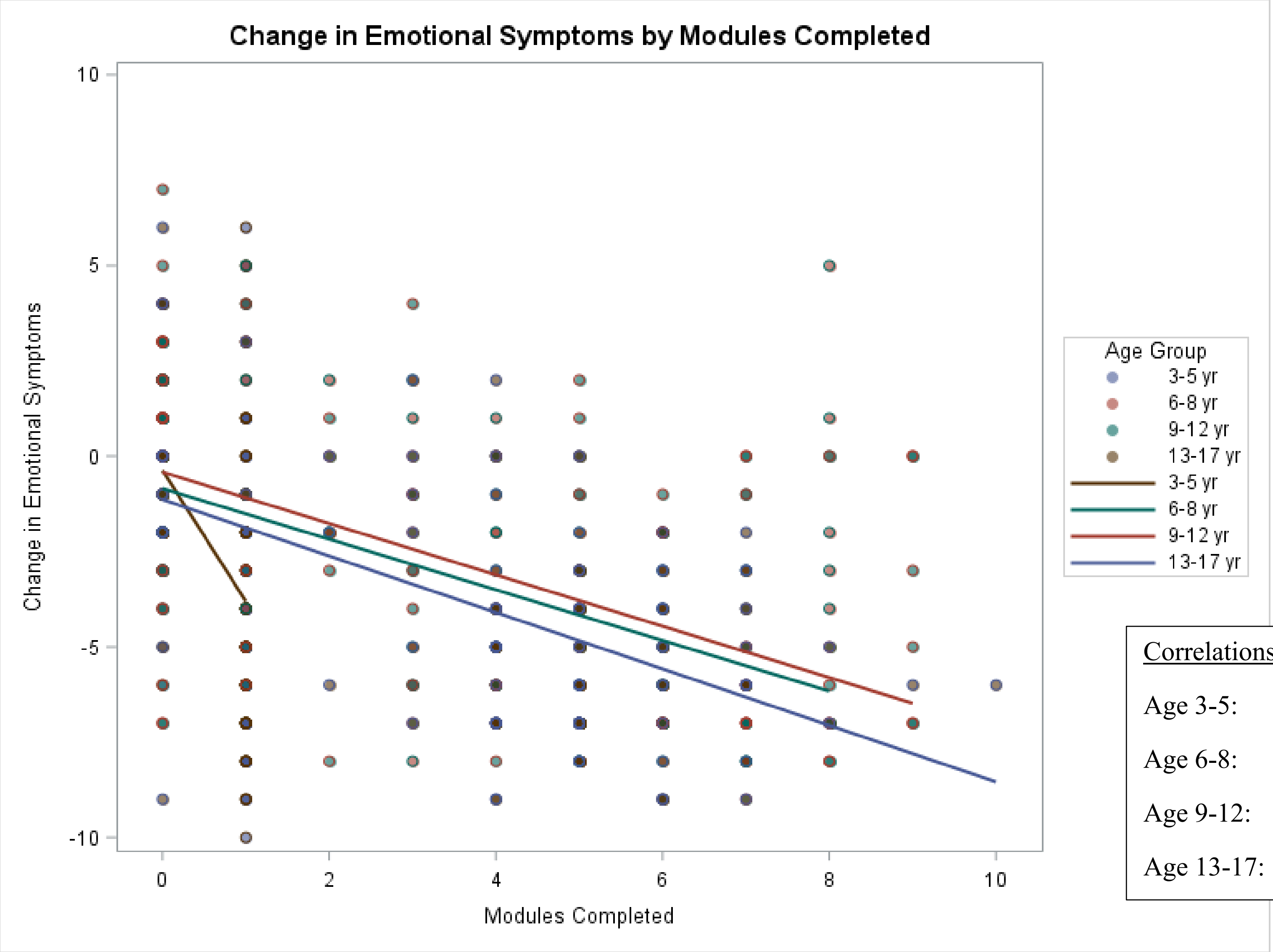

Note. Negative numbers indicate a lager improvement in symptoms from T1 to T3 (i.e., difference = T3 scores – T1 scores). This figure was generated in SAS proc sgplot. Please see the manuscript-specific OSF page (https://osf.io/wyu92) for a PDF of figures for all outcomes.

Correlations:	
Age 3-5:	*r* = -.34
Age 6-8:	*r* = -.52
Age 9-12:	*r* = -.55
Age 13-17:	*r* = -.54

Table 8. Predicting Number of Modules Used (i.e., intervention Engagement) from Baseline Sociodemographic Variables and Baseline Child Overall Well-being and impact of Problems, and Parent Mental Health.

Source	*b*	Chi-Square	*p*-value
SDQ total score	0.074	6.81	0.009
SDQ impact of problems	0.241	10.10	0.002
Intervention age group	--	1.12	0.572
Child gender	-0.030	0.01	0.906
Parent gender	0.457	2.27	0.132
Child age	-0.041	0.12	0.731
Parent age	-0.057	3.92	0.048
Parent income	0.244	4.98	0.026
Parent educational level	--	0.20	0.903
Parent depression symptoms	-0.066	0.34	0.559
Parent anxiety symptoms	0.009	0.01	0.933

Note. These analyses include those aged 6-17 (n = 340). We omitted those aged 3-5 years, as they only had one module. The unstandardized regression coefficient (*b*) and associated 95% CI come from Analysis of GEE Parameter Estimates, while the test statistics (chi-square and p-value) come from a Type 3 GEE Analysis. These tests are standard output from SAS proc genmod; model / type3 dist = normal. The unstandardized regression coefficients are only provided for continuous and binary predictors. For gender-variables, female is the reference group.

Table 9. Predicting Intervention Engagement (i.e., yes = 1 vs no = 0) from Baseline Sociodemographic Variables and Baseline Child Overall Well-being and Impact of Problems, and Parent Mental Health.

Source	*b*	Chi-Square	*p*-value
SDQ total score	0.000	0.00	0.996
SDQ impact of problems	0.398	3.19	0.074
Child gender	-0.686	1.06	0.303
Parent gender	0.682	0.51	0.475
Child age	-0.623	1.51	0.218
Parent age	0.183	4.48	0.034
Parent income	0.050	0.03	0.857
Parent educational level	--	2.60	0.272
Parent depression symptoms	-0.199	0.39	0.530
Parent anxiety symptoms	0.098	0.14	0.706

Note. These analyses include those aged 3-5 (n = 109). The unstandardized regression coefficient (b) comes from Analysis of GEE Parameter Estimates, while the test statistics (chi-square and p-value) come from a Type 3 GEE Analysis. These tests are standard output from SAS proc genmod; model / type3 dist = binomial (to obtain results for a logistic regression). The unstandardized regression coefficients are only provided for continuous and binary predictors. For gender-variables, female is the reference group.
